# Targeting tumor-associated macrophages with nanocarrier-based treatment for breast cancer: A step toward developing innovative anti-cancer therapeutics

**DOI:** 10.1016/j.heliyon.2024.e37217

**Published:** 2024-08-30

**Authors:** Ghazala Muteeb, Doaa S.R. Khafaga, Manar T. El-Morsy, Mohd Farhan, Mohammad Aatif, Mohamed Hosney

**Affiliations:** a Department of Nursing, College of Applied Medical Sciences, King Faisal University, Al-Ahsa, 31982, Saudi Arabia; b Health Sector, Faculty of Science, Galala University, New Galala City, 43511, Suez, Egypt; c Biotechnology Department, Faculty of Science, Cairo University, 12613, Giza, Egypt; d Department of Chemistry, College of Science, King Faisal University, Al Ahsa, 31982, Saudi Arabia; e Department of Basic Sciences, Preparatory Year Deanship, King Faisal University, Al Ahsa, 31982, Saudi Arabia; f Department of Public Health, College of Applied Medical Sciences, King Faisal University, Al-Ahsa, 31982, Saudi Arabia; g Zoology Department, Faculty of Science, Cairo University, 12613, Giza, Egypt

**Keywords:** Tumor associated macrophages, Breast cancer progression, M1, M2, Angiogenesis, Metastasis, Immunosuppression

## Abstract

Tumor-associated macrophages (TAMs) promote tumor advancement in many ways, such as inducing angiogenesis and the formation of new blood vessels that provide tumors with nourishment and oxygen. TAMs also facilitate tumor invasion and metastasis by secreting enzymes that degrade the extracellular matrix and generating pro-inflammatory cytokines that enhance the migration of tumor cells. TAMs also have a role in inhibiting the immune response against malignancies. To accomplish this, they release immunosuppressive cytokines such as IL-10, and TAMs can hinder the function of T cells and natural killer cells, which play crucial roles in the immune system's ability to combat cancer. The role of TAMs in breast cancer advancement is a complex and dynamic field of research. Therefore, TAMs are a highly favorable focus for innovative breast cancer treatments. This review presents an extensive overview of the correlation between TAMs and breast cancer development as well as its role in the tumor microenvironment (TME) shedding light on their impact on tumor advancement and immune evasion mechanisms. Notably, our study provides an innovative approach to employing nanomedicine approaches for targeted TAM therapy in breast cancer, providing an in-depth overview of recent advances in this emerging field.

## 1 Introduction

Tumor-associated macrophages are the most prominent immune cells found in malignancies [1]. TAMs constitute almost 50 % of the overall immune cells that infiltrate the tumor [1]. TAMs are commonly categorized into two distinct subgroups, known as M1 and M2 [2,3]. TAMs carry out a diverse array of roles through various phenotypes that are influenced by dynamic interactions, ultimately enhancing tumor growth [1,4]. The TAM1 phenotype is regarded as proinflammatory due to its ability to engage in phagocytosis and recognize cancer cells [5]. TAM1 induces tumor cell death while also promoting the production of proinflammatory cytokines, such as IL-12 and IFN-γ. TAM1 also expresses major histocompatibility complex (MHC) class II, which enhances their ability to display antigens on their surface [1,4]. On the other hand, the TAM2 phenotype has a function in reducing inflammation and is triggered by IL-13 or IL-4, leading to the generation of molecules that promote fibrosis. The prevalence of TAM2 increases, leading it to become the predominant subtype of TAMs in the tumor microenvironment [1]. TAM2 is recognized as a prominent “tumor promotor” that stimulates the development, invasion, and spread of tumor cells in breast cancer [4,6]. The inhibition of tumor growth was achieved by repolarizing TAMs into the TAM1 phenotype, taking advantage of the flexibility of TAMs [7]. An imbalance in the populations of M1 and M2 cells might lead to pathogenic occurrences [8,9]. An overabundance of M1 activation has been linked to the onset of chronic inflammatory disorders, whereas unregulated proliferation of M2 macrophages might result in significant immunological suppression [9,10].

Breast Cancer (BC) is highly frequent among women. Although surgical techniques and chemotherapy offer promise for recovery, the prognosis of BC deteriorates as the disease advances [11]. Different factors, including tumor grade, size, and nodal involvement, affect the prognosis of BC [11]. It has been confirmed that many molecular subtypes have unique characteristics and responses to treatment [12]. Additionally, a substantial association exists between an elevated level of TAM and various other factors, such as larger tumor size, basal phenotype, histologic grade, and blood vascular invasion [1,13]. Tumors can attract stromal cells such as, fibroblasts, immunological cells, and vascular cells by producing growth factors, cytokines, and chemokines. The recruiting process, in addition to the release of growth-promoting signals and tissue remodeling, plays a significant role in the development of TME, which has a substantial impact on tumor initiation, progression, metastasis, vascularization, and treatment responses [12].

Nanotechnology is essential for particularly targeting TAMs in the treatment of breast cancer [14]. Liposomes, polymeric nanoparticles, and micelles are types of nanocarriers that have been employed to transport chemotherapeutic agents and gene therapies to breast tumor cells [14,15]. This approach improves the effectiveness of treatment by increasing cytotoxicity and reducing the development of drug resistance [14,16]. In addition, researchers have created nanocarriers that respond to stimuli and can effectively target breast tumors. These nanocarriers are also capable of remodeling the tumor microenvironment and repolarizing macrophages to enhance antitumor immunity [15]. In addition, a versatile nanoplatform has been created to enhance the infiltration of T lymphocytes, eliminate tumor cells, and regulate immune responses by targeting PD-L1 and CD47 signals in breast cancer. This results in a more potent anti-tumor immune response and a shift of TAMs from protumor M2 like to antitumor M1 like phenotypes [17]. In summary, nanotechnology provides an opportunity to improve the effectiveness of breast cancer therapy by specifically targeting TAMs and regulating the immune response. Several treatment strategies primarily concentrate on specifically targeting cancer cells while disregarding the significant contribution of the TME in the formation and advancement of cancer [18]. In view of the evolving landscape of breast cancer research, our study stands out by focusing on targeted therapy for TAMs in the context of nanotechnology. We hope to transform breast cancer treatment by harnessing the unique features of nano carriers, providing a new perspective on TAM-targeted therapies. This review article not only bridges the gap between TAM biology and therapeutic innovation, but also develops the way for novel breast cancer treatment strategies that show significant promise for enhancing antitumor immune response and improving patient outcomes.

## 2 Methodological approaches

This review was conducted by performing a comprehensive search of the literature to gather information on the role of tumor-associated macrophages in breast cancer and the emerging nanomedicine approaches for targeted TAM therapy. The inclusion criteria for this review encompassed peer-reviewed original research articles, review articles and clinical trials that specifically focus on TAMs in the context of breast cancer, their mechanisms of promoting tumor progression, and therapeutic strategies targeting TAMs using nanotechnology. Studies involving human subjects, as well as *in vivo* and *in vitro* models, were included. Articles had to be published in English between 2004 and 2024 to ensure the inclusion of the most recent and relevant research. The exclusion criteria eliminated studies that did not directly address TAMs in breast cancer, non-English publications, duplicate studies, and non-research articles such as editorials and commentaries. Databases searched included PubMed, Web of Science, Scopus, and Google Scholar using keywords such as tumor-associated macrophages, breast cancer, tumor microenvironment, angiogenesis, nanomedicine, and targeted therapy. Data extraction focused on study design, population, key findings related to TAMs and breast cancer, therapeutic strategies, and outcomes. The quality of included studies was assessed using standardized criteria appropriate for the type of research.

## 3 Breast cancer

Worldwide, breast cancer is the most prevalent form of cancer and is recognized as a major contributor to cancer-related deaths in developing as well as developed countries. Approximately 2.3 million women globally received a breast cancer diagnosis in 2020, resulting in 685,000 fatalities. By the year 2023, the United States is projected to see the diagnosis of breast cancer in about 300,000 women. Approximately 20 % of individuals who are newly diagnosed with breast cancer have a familial history of the disease [19]. The prevalence of breast cancer continues to be a major worldwide health issue, requiring the creation of creative and efficient treatment approaches. Breast cancer is a prevalent malignancy in women and may also affect males, but less often. Recognizing the many subtypes of breast cancer is crucial for adapting therapy strategies for individual patients. This information may be useful in achieving a correct diagnosis, designing treatment strategies, and improving patient prognosis [20]. Multiple studies have demonstrated that various factors, such as age, race, socioeconomic status, genetic factors like BRCA mutations, hormonal factors like age at menarche, parity, age at first full-term pregnancy, breastfeeding, and lifestyle-related factors like diet, physical activity, alcohol use, and tobacco use, are all linked to a higher risk of breast cancer [21].

## 4 Macrophages in breast cancer

Macrophages, a type of white blood cell responsible for phagocytosis, play a vital role in the human immune system [18,22]. They contribute to both innate and cellular immunity [22]. Originating from monocytes, which differentiate from precursor cells in the bone marrow [7,22]. TAMs possess a variety of types, including conventional types of M1 and M2 cells, as well as TCR^+^ and CD169^+^ macrophages [23]. Initially, TAMs were mistakenly classified as M2 cells due to their similar abundance around the tumor, secretion of various cytokines, and promotion of metastasis in breast cancer cells [23,24]. However, unlike M2 cells, TAMs do express INOS and the CD200 receptor [25]. Macrophages can follow two activation pathways upon exiting blood vessels, resulting in the formation of M1 or M2 macrophages [15,26]. These macrophage subtypes exhibit distinct characteristics and immunophenotypes. Notably, the NF-kB signaling pathway participates in the polarization of M1 cells, while M2 cells do not rely on this pathway [27]. M1 macrophages produce pro-inflammatory cytokines, possess strong antigen-presenting abilities, and promote immune responses against bacteria and tumor cells [28]. Conversely, M2 macrophages primarily release immunosuppressive cytokines, have limited antigen-presentation capabilities, and contribute to tissue repair, wound healing, and blood vessel formation [10,28]. Traditionally, macrophages have been recognized as phagocytes and eliminators of tumor cells, aiding in cancer cell clearance [29]. Macrophages are a major component of immune infiltration in breast cancer cells [30]. However, in breast cancer patients, macrophages prefer to accumulate in malignant tissues and their environs. This buildup leads to the release of reduced immune factors, enhanced osteolysis, promotion of lymphatic metastasis, and resistance to radiation and chemotherapy, promoting tumor growth and enabling recurrence, particularly in patients with negative prognoses [31]. Breast cancer is one of the most commonly diagnosed cancers and the leading cause of cancer-related deaths in women worldwide [32]. Breast cancer cells and their microenvironment have been shown in recent studies to eliminate the production of inflammatory response factors in breast cancer and surrounding tissue [5]. As a result, M2 macrophages aggregate, and M0/M1 macrophages convert into M2 macrophages, culminating in the growth of TAMs [4,33].

## 5 Macrophage activation and mechanism of action

**Fig. 1:**
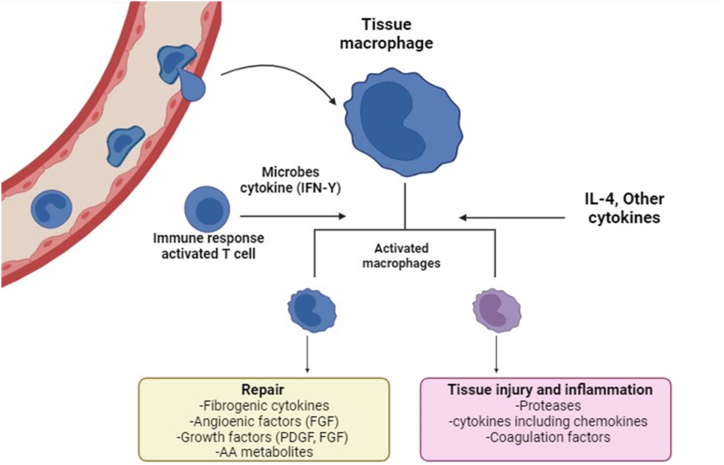
Activation of tissue macrophage. Microbes cytokine (IFNγ), fibroblast growth factors (FGF), platelet derived growth factor (PDGF)and Interleukin 4 (IL-4).

Through Fcγ receptors on the surface of tumor cells, like CD20 and calreticulin, M1 macrophages may recognize and target tumor cells, which facilitates the phagocytosis of tumor cells by M1 macrophages [16,34,35]. Additionally, M1 macrophages stimulate T cells, improve the expression of CD4 and CD8, induce immune responses against tumor cells, and inhibit tumor formation [34]. These “eat me” signal molecules are specific to cancer cells and are absent in normal cells. They are essential for the balance of “eat me” and “don't eat me” signals when macrophages are phagocytosing breast cancer cells [5]. Macrophages are capable of specifically phagocytosing malignant cells while protecting normal cells by blocking the CD47-SIRP signaling pathway [34,36]. Nevertheless, recent research has shown that enhancing macrophage-mediated anti-tumor activity requires more than just lowering inhibitory signals like CD47 [34]. Two signals are necessary for the effective activation of macrophages. First, there needs to be an activation signal a Toll-like receptor agonist, for example. Secondly, an additional signal, such as a CD47 inhibitor, is needed to reduce the threshold for anti-cancer effects [37]. Expanding on this observation, CPG, a Toll-like receptor agonist, was utilized to promote macrophages, initiating the first signal. TME has been found to foster tumor development, but the presence of M1 cells via the TME mitigates the TME's impact on breast tumors. Conversely, the presence of M2 cells promotes tumor growth. Therefore, reprogramming M2 macrophages into the M1 phenotype is imperative [38]. The utilization of cGAMP-NP has been shown to enhance the expression of MHC and co-stimulatory molecules, elevate CD4^+^ and CD8^+^ expression, and result in T cell activation by stimulating IFNγ through STING, as illustrated in Fig. 1.

Consequently, this results in the eradication of breast cancer tumors and facilitates the conversion of M2 macrophages into the M1 phenotype [39]. Furthermore, the cationic polysaccharide spermine pullulan has exhibited the ability to augment the function of TLR1, TLR2, and TLR3 receptors, promote the ERK pathway, increase CD4 and CD8 levels in tissues, and decrease CD31 expression, thereby promoting the polarization of M0/M2 macrophages toward the anticancer M1 phenotype [40]. Further, it was recently shown that an extract made from the herb combination of Scutellaria barbata and Hedyotis difusa upregulates the expression of miR155 in M2 macrophages while downregulating it in M1 macrophages. Additionally, YDW11 prevents breast cancer cells from migrating by inhibiting macrophage polarization toward the M2 phenotype [40].

## 6 TAMs as modulators of the breast cancer tumor microenvironment

TAMs have received considerable attention in cancer research, and scientists are currently working on them as a cancer therapeutic target. TAMs gather in large quantities in the vicinity of tumors and have a substantial impact on cancer growth, leading to their being labeled “traitors” [41]. Thus, M2 cells aid in the restructuring of blood vessels and the formation of lymphatic channels, both of which contribute to the progression of cancer [24,37]. Furthermore, tumor cells actively enhance M2 cell synthesis and recruit more M2 cells to the tumor location [24,42]. They also produce additional cytokines that support M0 cell transformation into TAMs and control certain microRNAs, ERK, P53, and other various signaling pathways that promote M1 to M2 cell conversion [43].

## 7 Role of TAMs in breast cancer development

**Fig. 2:**
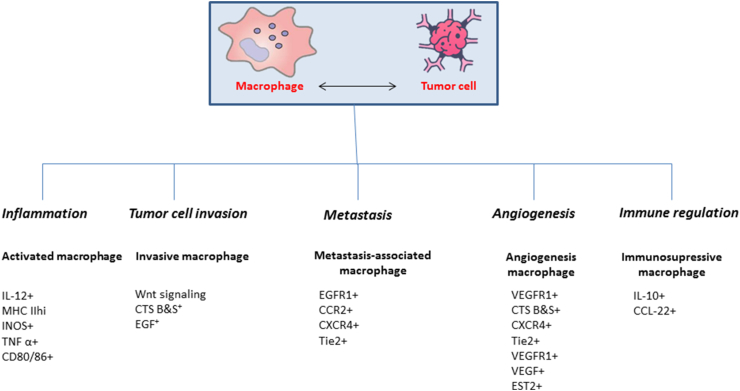
The role of tumor-associated macrophages (TAM) in cancer cell progression.

TAMs encompass a heterogeneous population of cells with diverse functional effects in both steady-state and pathological conditions. The composition and behavior of TAMs are regulated by various mechanisms, including soluble factors derived from tumor cells, alterations in tumor metabolism, interactions with other immune cells, and additional factors [44]. While bone marrow monocytes are the primary source of TAMs in tumors, current evidence indicates that the recruitment of circulating inflammatory monocytes is critical for TAM accumulation, which is controlled by TME factors such as chemokines and cytokines [45]. Monocytes originating from bone marrow form long-lived TAMs within the TME. TAMs can be found in both hypoxic and normoxic regions of the tumor [46]. Importantly, TAMs not only adapt to their TME location but also reciprocally shape the composition of the surrounding TME [47]. The localization and extent of TAM infiltration pose challenges for targeted interventions in breast tumors [47]. TAMs predominantly exhibit an M2 phenotype, often referred to as pro-tumor, as they facilitate cancer cell proliferation, angiogenesis, and metastasis through distinct anti-inflammatory mechanisms [48,49] as illustrated in Fig. 2.

It is worth noting that not all inflammation is beneficial during homeostasis, as prolonged inflammation can promote malignant cell transformation and tumor growth [24,36]. TAMs enhance tumor progression by releasing growth factors, such as epidermal growth factor receptor (EGFR), which result in breast cancer proliferation [39]. Human breast cancer cell lines and animal models illustrate that breast tumor cells can produce colony-stimulating factor (CSF)-1 (macrophage [M]-CSF) and CCL2, which are considered the most significant attractants and growth factors for TAMs. CSF-1 and its receptor (CSF-1R) assist in the regulation of macrophages; CSF-1R and/or CSF-1 expressions are potentially associated with a poor breast cancer prognosis [50]. The invasive capacity is increased by the production of CSF-1 from cancer cells [51]. CSF-1 stimulation not only increases TAM recruitment but also results in the development of a large density of vascular networks that promote tumor growth [51].

### 7.1 TAMs promote tumor invasion and metastasis

Cells with pleiotropic functions release exosomes, which are small lipid bilayer particles that are typically differentiated based on size and markers [52]. Previous studies reported that pleiotropic cells regulate the intracellular pathway involved in all stages of breast tumor development until metastasis dissemination [52]. Both TAMs and tumor cells undergo alterations in cellular metabolism, which shape their functional phenotype in a mutually influencing way [53,54]. TAMs can regulate multiple mechanisms associated with the dissemination of carcinoma cells. For instance, TAMs secrete proteolytic enzymes such as matrix metalloproteinases-2 and 9 (MMP-2 and MMP-9) that can degrade components of the basement membrane, thereby facilitating tumor cell intravasation and spreading in blood and lymphatic vessels [55]. Many studies have demonstrated that tumor-derived exosomes have a significant function in transforming monocyte-derived macrophages into regulatory macrophages [4]. Necrosis is another feature of tumors that is related to both the poorprognosis and the presence of TAMs [56]. Tumors with necrosis-state have a hypoxia area that attracts TAMs, in addition to the polarization of TAMs towards the M2 phenotype by CSF-1 expression and the production of factors that assist in the promotion of tumors and metastasis [56].

### 7.2 TAMs promote angiogenesis

TAMs enhance angiogenesis in breast cancer cells. TAMs are a rich supply of pro-angiogenic factors, such as SPARC, VEGF, chitinase like proteins, the SEMA and S100A families, and osteopontin [57]. M2 subtypes that are recruited and accumulated within TME play a significant role in angiogenesis by producing proangiogenic factors like vascular endothelial growth factor (VEGF), platelet-derived growth factor (PDGF), and fibroblast growth factor (FGF) [43,57]. These factors enhance blood vessel formation in tumors, which is crucial for facilitating tumor development and metastasis [26]. Small protein molecules can regulate leucocyte transportation, called chemokines, in response to homeostasis or inflammation [58,59]. TAMs and their pro-angiogenic activities may be targeted as a potential treatment approach for breast cancer to reduce tumor angiogenesis and inhibit tumor growth [[60], [61]]. CXCL18 is one of the major chemokines that accumulate in TAMs in breast cancer [62]. CXCL18 promotes tumor growth and invasion in breast cancer by inducing IL-4, IL-13, and IL-10 in TAMs [62]. The direct binding between chemokines and chemokines receptors changes the expression of secondary messengers to promote angiogenesis and inhibit the infiltration of the antitumor cells [63]. Nanocarriers are essential in reducing the development of breast cancer by specifically inhibiting angiogenesis and TAMs [64,65]. Research has demonstrated that nanoplatforms such as Boltorn® H40-PEG-MTX-anti-VEGFR2 nanobodies can impede the movement, infiltration, and growth of cells, thereby effectively restraining the development of blood vessels near cancerous cells and impeding the advancement of tumors [65,66]. In addition, lipopolymeric hybrid nanostructures with spermine tethers have demonstrated targeted uptake by breast cancer cells. This uptake triggers apoptosis and exerts a potent antiangiogenic effect by influencing the VEGF pathway, cell proliferation, invasion, and migration. These findings demonstrate the potential of nanocarriers in combination chemotherapy for effectively inhibiting the progression of breast cancer [67]. Nanotechnology in drug delivery systems shows potential for improving the antiangiogenic effects of breast cancer treatment through precise targeting of tumor cells and their microenvironment.

### 7.3 TAMs suppress the immune system

TAMs play a role in modulating the effectiveness of T cells and natural killer (NK) cells in eliminating tumor cells. In cases of liver fibrosis, M1 macrophages enhance the population of activated NK cells, release TNF related apoptosis inducing ligand (TRAIL), and induce apoptosis in cancer cells [15]. In mesothelioma, M2 macrophages in pleural effusion show an inverse correlation with T cells *in vivo*, indicating the potential for targeting macrophages in mesothelioma treatment [68]. Furthermore, TAMs can directly hinder the proliferation of CD8^+^ T cells through various mechanisms, including arginase- 1 metabolism of L-arginine, iNOS, oxygen radicals, or nitrogen species [59]. TAMs also recruit regulatory T cells (Tregs) through CCL22 [69]. Further suppressing the immune response of T cells against tumors. Experimental removal of TAMs impedes Treg cell recruitment, restrains tumor growth, and reduces the level of CCL20 in mice with xenograft tumors [70,71]. Extensive evidence suggests that inflammation at the tumor site can promote tumor progression. Inflammation and immune evasion are recognized as critical features of cancer. TAMs contribute to cancer related inflammation by promoting the generation of inflammatory T-helper subsets, such as TFH. Toll-like receptor 4 (TLR4) induced inflammation in monocytes plays a crucial role in inducing IL-21+ TFH like cells [2,62]. Through IL-21-IFNγ-dependent pathways, they facilitate plasma cell differentiation and create a favorable environment for M2b macrophages and cancer progression [71,72]. Furthermore, TME can undergo metabolic changes that can also contribute to the immunosuppressive nature of TAMs. Alterations in food availability and metabolic pathways can shift macrophage polarization towards a protumor phenotype, decreasing their capacity to generate an active anticancer immune response [71]. These findings suggest that strategies aimed at influencing the functional activities of inflammatory cells may hold therapeutic benefits in cancer treatment.

## 8 Targeting pathways for breast cancer suppression

Multiple previous studies have provided evidence of frequent mutations in the Ras gene in human cancer. In breast cancer, elevated expression and activation of Ras have been associated with increased aggressiveness of tumors, suggesting its potential as a prognostic indicator for breast cancer progression [73]. The RAS/RAF/MAPK signaling pathway plays a critical role in regulating cellular processes such as proliferation, survival, and apoptosis [74]. Activation of this pathway can result in the downregulation of PAR4, a tumor suppressor protein that specifically promotes apoptosis in cancer cells [75]. Downregulation of PAR4 has been observed in various cancer types, including breast cancer [74,76]. Silencing PAR4 using RNA interference (RNAi) has been demonstrated to enhance the proliferation of MCF-7 cells. Conversely, increased expression of PAR4 has been shown to reduce the proliferation rate of MCF-7 cells and enhance their sensitivity to docetaxel, a chemotherapy drug [77]. One of the attractive targets for breast cancer treatment is macrophages, which are considered independent cofactors in breast cancer [58]. There are various strategies based on enhancing tumor therapy via TAMs.

### 8.1 TAMs depletion

One appealing treatment strategy for reducing tumor development and drug resistance is progenitor depletion, also known as TAMs [37,[78], [79], [80]]. Chemotherapeutic drugs, such as doxorubicin and docetaxel, can also stop the growth of tumors in breast cancer by eliminating TAMs [58,64]. Administering monoclonal antibodies coupled with immunotoxins that target antigens expressed by TAMs is an additional approach [78,81]. Bisphosphonate compounds are taken up by macrophages to inhibit their proliferation and induce apoptosis in cancer cells [82,83]. Studies have demonstrated that RNA aptamers and trabectedin can specifically eliminate tumor-associated macrophages and trigger caspase-8-mediated apoptosis through TRAIL receptors, respectively [84]. However, M2pep, which includes a proapoptotic peptide, has been discovered to target and eliminate TAMs, leading to improved survival rates in mice with tumors [85]. Providing the proapoptotic peptide to TAMs alone delayed death and selectively decreased the M2 like TAM population [44].

### 8.2 Macrophage disruption

Chemokines and CSF1 have important functions in attracting and regulating monocytes within tumors. They play a crucial role in decreasing the release of monocytes from the bone marrow by targeting the CSF1-CSF1R and CCL2-CCR2 signals [15]. Consequently, the infiltration of precursor cells and the differentiation of macrophages are diminished in mammary tumors and areas where metastasis may occur [7,86]. In experimental models of breast cancer, inhibitors of CSF1R signaling have been shown to enhance the effectiveness of chemotherapy or radiotherapy by suppressing tumor growth and the spread of cancer to other parts of the body [15,87].

### 8.3 TAM reprogramming

M1 and M2 macrophage polarization involve many signaling pathways [27,48]. TAMs are repolarized towards TAM2, which is a tumor inhibitor and proinflammatory [88]. The antiinflammatory cytokine IL-10 can be mediated by STAT3, which has the potential to be employed in the process of reprogramming macrophages from the M1 to M2 phenotype [89]. In cancer, miRNAs have a crucial role in regulating TAM phenotypes by inducing M2 like polarization and preventing CD8^+^ cytotoxic T lymphocytes from infiltrating tumors [89]. Zoledronic acid (ZA) can also decrease the frequency of tumor-associated macrophages and reverse their polarization from M2 to M1, hence reducing spontaneous mammary carcinogenesis [82,90].

## 9 Therapeutic agents targeting TAMs

### 9.1 Small molecule inhibitors and immunomodulators for TAM modulation

Recent studies focused on investigating small molecule inhibitors and immunomodulators to regulate TAMs in the TME [91,92]. TAMs play a significant role in enhancing cancer growth and immune suppression, thereby leading to immune checkpoint inhibitor (ICI) resistance [59]. In preclinical mouse models, inhibition of TAM signaling pathways via a small molecule inhibitor has demonstrated its effectiveness [93]. Among the most promising types of small molecule inhibitors for TAM modulation are colony stimulating factor-1 receptor (CSF-1R) inhibitors and focal adhesion kinase (FAK) inhibitors [91]. By reprogramming the TME and TAMs, CSF-1R inhibitors enhance the eradication of breast cancer by T-cell-mediated mechanisms [92,94]. Furthermore, FAK inhibitors alter TME to enhance an antitumor immune response and reduce the invasion of immunosuppressive cells, such as TAMs [3]. The combination of small-molecule inhibitors with ICI presents an opportunity to enhance the efficacy of immunotherapy by specifically targeting TAMs.

### 9.2 MicroRNA-based therapeutics for TAM targeting

MicroRNA-based therapies for breast cancer have shown promising results, especially when paired with immune checkpoint inhibitors (ICIs) [36]. Furthermore, miRNAs actively contribute to the advancement of breast cancer by affecting stem cell production, initiation, invasion, metastasis, and angiogenesis [95]. Researchers have studied various delivery technologies, such as nanoparticles, liposomes, and viral vectors, to efficiently target breast cancer cells for miRNA delivery [96]. Developing techniques that target dysregulated miRNAs in TAMs could aid in the remodeling of TME [96,97]. Several miRNAs have been discovered that target macrophage genes as well as influence TAM polarization [97]. MiR-155, for example, is a microRNA that has been found to target TAMs in breast cancer. It can block TAM- M2 -like polarization and stimulate an anticancer immune response [98]. A different approach to microRNA that can be targeted in TAMs in TNBC is miR-146a. It inhibits TAM protumor activities while enhancing the antitumor immune response [98]. Targeting TAMs using microRNAs can potentially improve the efficacy of breast cancer treatment by modulating the tumor microenvironment and enhancing the antitumor immune response. Further research and development of these therapies may lead to improved treatment outcomes for breast cancer patients.

### 9.3 Antibody-based therapies for TAM depletion or reprogramming

In recent years, antibody-based therapies have demonstrated clinical efficacy and safety as a treatment for breast cancer [13]. Monoclonal antibodies (mAbs) have recently been approved as cancer therapies, with remarkable effectiveness [13,95]. Antibody-based treatments targeting TAM depletion or reprogramming in breast cancer have shown progress in recent years [99,100]. TAM depletion has been found to generate a favorable environment for delivering antiprogrammed cell death protein 1 (PD-1) antibodies, hence improving immunotherapeutic efficacy [94]. On the other hand, some targeted therapeutic techniques, such as the use of toxin-conjugated monoclonal antibodies, entail the depletion of M2 [7,71]. Therefore, antibody-based therapeutics can target and treat breast cancer via TAM depletion or reprogramming, hence providing novel tools for therapy.

## 10 TAMs and drug resistance

In breast cancer treatment, the most difficult challenge is drug resistance [101]. According to recent studies, TAMs are associated with drug resistance [102]. TAMs are a crucial component of TME, and their impact on breast cancer chemotherapeutic resistance cannot be neglected [101]. TME consists of numerous cell types, extracellular matrix, and soluble substances. It has a significant impact on treatment efficacy, which is referred to as environmental mediated drug resistance (EM-DR) [103]. TAMs are one of TIME, which are critical for drug resistance via metabolic reprogramming to induce tumor angiogenesis [101]. As well as the secretion of inflammatory cytokines and chemokines such as TNF-α, IL-6, IL-10, and CCL18 [101]. Secretion of IL-10, which is responsible for the regulation of BCL-2 and STAT3 expressions, induces the activation of the IL-10-STAT3-BCL2 pathway in breast cancer, which enhances drug resistance [71]. TAMs cause immune therapy resistance by suppressing the functions of T-cells [101]. Doxorubicin (DOX) is a chemotherapeutic drug that is frequently used to treat breast cancer [101]. TAMs can increase the level of FABP5 and PPAR in malignant breast cells, activating the CaMKII signaling pathway and leading to DOX resistance [5,104]. TAMs can cause a buildup of ATP binding cassette (ABC) transporters, which are proteins that eliminate DOX from breast cancer cells, resulting in DOX resistance [81]. Furthermore, combining TAM-targeted medicines with conventional chemotherapeutics can help improve the prognosis [105]. Overall, TAMs could affect the response of drugs to various types of cancer, and targeting these types of cells could be a powerful approach to reduce drug resistance and increase therapy effectiveness.

## 11 Nanoimmunotherapies approaches of TAM

**Fig. 3:**
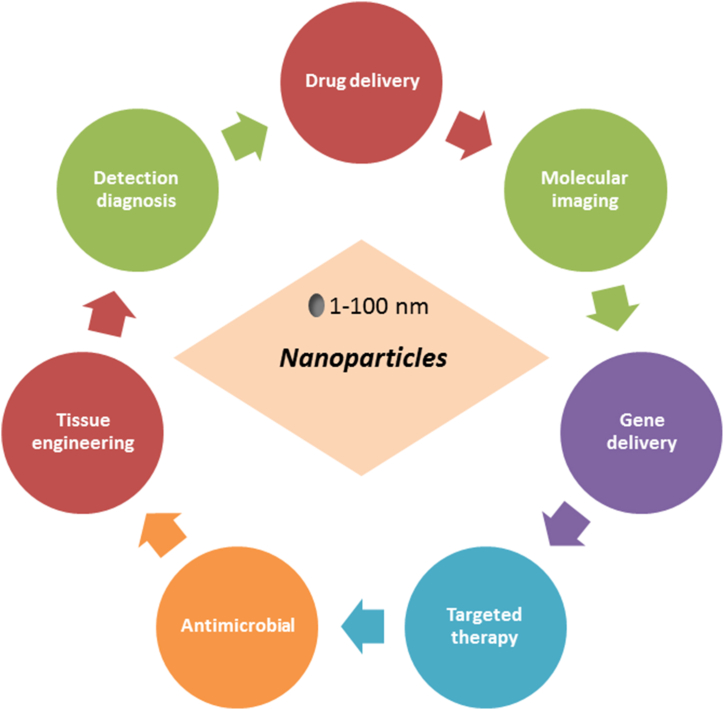
Several applications of nanoparticles in biomedical field.

Nanotechnology is an interdisciplinary scientific field that encompasses various types of nanoparticles and novel nanodevices used in diverse areas of research [106,107]. Its application in tumor diagnosis aims to detect malignancies at an early stage, reducing the number of patients with advanced cancer [108,109]. Nanotechnology research and technology present advancements in various fields such as materials and manufacturing, nanoelectronics, healthcare and medicine, energy, biotechnology, information technology, and national security [105,109]. As illustrated in Fig. 3, nanoparticles have been utilized in various biomedical applications. Notably, their potential as a targeted delivery system for cancer cells represents a significant enhancement over existing breast cancer therapies and imaging tools [106,110].

Recent advancements in nanotechnology have allowed for the modification of nanostructures to selectively bind to tumor-specific receptors, thereby enhancing diagnostic accuracy and sensitivity [80]. Nanomaterial-based contrast agents, such as ultrasmall superparamagnetic iron oxide (USPIO) and superparamagnetic iron oxide (SPIO), exhibit prolonged circulation in the bloodstream and can target specific cell surface markers, leading to improved contrast properties in MRI imaging and facilitating reliable tumor diagnosis [59,94]. Tailored nanoparticles have shown promise in targeted chemotherapy, delivering drugs specifically to tumors while minimizing toxicity to surrounding normal cells, thereby enhancing the effectiveness of radiotherapy and improving treatment outcomes [111]. The modifiable characteristics of nanoparticles, including size, shape, charge, surface properties, and functionality, can be synergistically employed with precision medicine approaches to optimize patient stratification methods, indicating the potential of nanoparticles in the era of precision medicine [80,87,112]. Nano immunotherapies, utilizing nanomaterial-based formulations, have the potential to enhance the therapeutic benefits of immunotherapies by targeting the immunosuppressive tumor microenvironment and activating the immune system through interactions with other immune cells. Recent advancements in nanobiotechnology have sparked interest in utilizing nanomaterials for tumor immunotherapy due to their targeted drug delivery, controlled release at specific locations, surface functionalization capabilities, conjugated therapy potential, and low immunogenicity, resulting in effective immune system activation [87,113]. Specifically, tailored drug delivery methods involving a range of nanomaterials have revolutionized TAM related immunotherapies [93,114]. These nanomaterials may improve the efficacy of immunotherapies by targeting the immunosuppressive microenvironment, interacting with immune cells, and reducing off-target toxicity and immune-related adverse effects. Studies have shown that nanoparticles may trigger the repolarization of antiinflammatory M2 type macrophages towards a proinflammatory M1 phenotype, leading to tumor inhibition in several malignancies [14,92]. TAMs are further discovered to act as drug accumulation reservoirs, facilitating local delivery of nanotherapeutics to tumor cells and enhancing their efficacy by changing the spatial distribution of pharmacological chemicals within tumors. Notably, tumor macrophage uptake of nanoparticles is critical to generating therapeutically beneficial drug accumulation within tumors, as the elimination of macrophages significantly reduces nanoparticle deposition and renders the treatment ineffective [115]. TAM-targeting nanoimmunotherapies have become beneficial by combining the synergistic effects of TAMs and nanomaterials, and many macrophage-targeting nanomedicines have been developed in recent years.

### 11.1 Various nanomaterials for TAMs focused immunotherapy

**Fig. 4:**
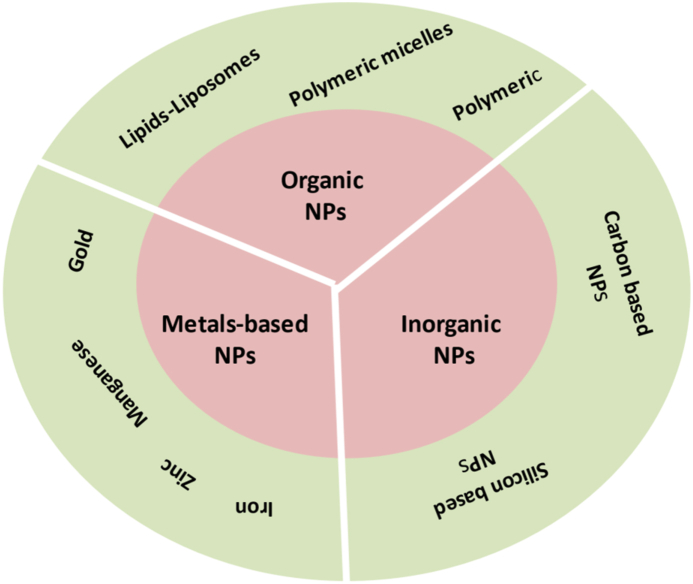
Different types of nanoparticles (NPs) are used in drug delivery systems.

As illustrated in Fig. 4, there are currently several types of nanoparticles (NPs) available for drug delivery in TAM-centered cancer immunotherapy [15,113,116]. Organic nanomaterials (such as lipids/liposomes, polymeric micelles, and polymeric NPs), inorganic nanomaterials (such as carbon based NPs, silicon-based NPs, and metal-based NPs such as gold, manganese, zinc, and iron) [15,87,117], as shown in Table 1.

**Table 1:** Advantages and disadvantages of various nanocarriers.

Nanocarrier	Classification	Advantages	Disadvantages	Ref.
Silicon NPs	Inorganic	Biocompatible, nontoxic, photoluminescence, and highly adsorbent	Based on the size and dosage, it has the potential to induce cytotoxicity, oxidative stress, and DNA damage	[131,132]
Carbon NPs	Inorganic	Unique optical, electrical, mechanical, thermal, chemical characteristics, and have antibacterial and antiinflammatory properties	Induce oxidative stress	[133,134]
Ceramic NPs	Inorganic	CNs NPs have dielectric, optical, magnetism characteristics and show multiple bioactive characteristics, including antibacterial activity, tissue regeneration, and wound healing	Cell damage, genotoxicity and induce oxidative stress	[[135], [136], [137]]
Lipid NPs	Organic	Low toxicity, biocompatibility, and excellent control of drug release	Minimal drug loading capacity	[138]
Polymeric micelles	Organic	High stability in physiological media, the ability to decorate surfaces with targeted ligands, and Increased permeability and retention effect for passive tumor targeting	Synthetic polymers may cause toxicity, and immunogenicity challenges	[139]
Dendrimers	Organic	Highly branched, monodisperse, biocompatibility, high permeability, polyvalency, chemical stability, and low immunogenicity	Intrinsic toxicity	[140,141]
Gold NPs	Metals	Remarkable optical, electrical, and photothermal characteristics for imaging and targeting therapy	The tendency to aggregate results in a decrease in bioavailability and Potential cytotoxicity and genotoxicity at high dosages	[142]
Metal oxide NPs	Metals	Superparamagnetic characteristics enable magnetic targeting and MRI imaging	Potential toxicity and induces oxidative stress in cells and organs	[143,144]

These NP formulations can efficiently carry and deliver drugs to TAMs. As previously mentioned, NPs have a higher tendency for internalization by TAMs, making them suitable drug carriers for macrophages for targeting tumor cells. Furthermore, when NPs are engineered to target and phagocytosis TAMs, the physical properties of NPs, such as size, charge, and shape, can influence the phagocytosis response. Therefore, the selection of appropriate carrier materials is crucial for the success of TAM targeting immunotherapy.

#### 11.1.1 Inorganic nanocarriers for breast cancer therapy

Inorganic nanocarriers are essential in breast cancer treatment as they effectively manipulate the tumor microenvironment, which includes their ability to target TAMs [118]. Nanocarriers, such as silicon, carbon-based, ceramic, and up-conversion nanoparticles, have demonstrated notable benefits in photodynamic therapy (PDT) for breast cancer treatment. These benefits include improved solubility, biodistribution, and intercellular penetration, resulting in more efficient destruction of cancer cells [119]. In addition, inorganic nanoparticles have been employed in drug delivery systems to enhance targeting, mitigate tumor resistance, and augment the effectiveness of chemotherapeutic drugs such as doxorubicin in breast cancer chemoimmunotherapy [118,120]. Incorporating inorganic nanocarriers shows great promise in addressing immunosuppressive circumstances in the tumor microenvironment and augmenting the antitumor immune response. Consequently, they serve as effective tools for combating breast cancer.

#### 11.1.2 Organic nanocarriers for breast cancer therapy

Organic nanocarriers with specific physicochemical features and surface modifications, such as lipid-based nanocarriers, polymeric micelles, dendrimers, and polymeric nanoparticles, can be engineered to penetrate solid tumors and target TAMs [114,[121], [122], [123]]. To enhance the therapeutic efficiency of breast cancer treatments by targeting TAMs, polymer-based nanocarriers such as dendrimers, polymeric nanoparticles, and polymer micelles have grown in recent years [122]. To improve TAM targeting, scientists can change the surface of liposomes and liquid crystalline nanoparticles (LCNPs) by adding cationic chitosan and anionic hyaluronic acid. Researchers found that tamoxifen and resveratrol-loaded TAM targeting LCNPs significantly reduced the growth of breast cancer cells [122].For instance, docetaxel-based self-emulsified drug delivery systems (SEDDS) have shown promise in identifying and removing TAMs from breast cancer cells [14,122]. Overall, the incorporation of therapeutic medicines into organic nanocarriers. This nanoformulation can enhance breast cancer patient s' treatment efficacy. Organic nanocarriers for TAM targeted breast cancer treatment have promise but require more study and clinical trials to confirm their efficacy.

#### 11.1.3 Metal nanocarriers for breast cancer therapy

Metal nanocarriers have a vital role in breast cancer treatment, particularly to target TAMs. Nanocarriers, such as gold nanoparticles and metal oxide nanoparticles, possess distinct characteristics that improve the distribution of drugs and the effectiveness of therapy in the treatment of breast cancer [124,125]. Functionalizing them with biomolecules enhances their selectivity against cancer cells, triggers cytotoxic effects through ROS production and apoptosis, and improves the absorption of drugs by breast cancer cells. As a result, their cytotoxicity significantly increases compared to free medicines [126,127]. Evidence suggests that gold nanoparticles may be useful in the delivery of chemotherapy drugs such as DOX to patients undergoing treatment for breast cancer [128]. Another approach is based on applying iron oxide nanoparticles; researchers were able to construct designed nanocomplexes that limit the polarization of TAMs to the protumor M2 type, presenting a unique approach to TAM treatment in breast cancer [129]. To further enhance anticancer effects through alterations to the tumor microenvironment, metal complexes integrated into dual target nanotherapeutics have demonstrated remarkable cytotoxic and immunomodulatory capabilities [130]. Moreover, metal nanocarriers can modify the tumor microenvironment, reorient macrophages to enhance the immune response against tumors, and specifically inhibit the growth of breast tumors. These properties make them highly promising in the battle against breast cancer, particularly in dealing with TAMs [127].

## 12 Nanotechnology approaches of TAM specific drug delivery for breast cancer

Nanomaterials can be designed to deliver drugs into the tumor region via passive or active mechanisms. Active targeting requires binding components such as antibodies and peptides to receptor structures expressed in the target region [145]. In passive targeting, the drug carrier complex generates circulation via the circulation and is driven to the target site by affinity or binding regulated by variables such as pH, molecular site, temperature, and shape [14]. The principal targets of the body are cell membrane receptors, lipid membrane components, and cell surface antigens or proteins. Nanotechnology-enhanced drug delivery systems are currently being developed to prevent and treat malignancies [146]. Drug delivery system (DDS) advancements have resulted in improved properties such as reduced particle size, higher permeability, increased solubility, and improved effectiveness [147]. These enhancements have made drug delivery more selective, precise, and focused, effectively addressing challenges associated with certain treatment classes [148,149]. Numerous therapeutic approaches and conventional diagnostic strategies have been investigated to improve accuracy in diagnosis and drug specificity with the recent development of nanomedicine, drug discovery and design, and drug delivery systems [148]. For example, novel pharmaceutical delivery systems are being investigated with a view to ensuring targeted action, minimizing toxicity, and enhancing bioavailability [148]. Despite these advances, there are still challenges with drug delivery, such as targeting drugs to tumor locations and achieving continuous release through a specified time [147]. To overcome these concerns, researchers are focused on the development of multifunctional delivery systems and the combination of diverse drug delivery mechanisms to ensure optimal drug distribution and desired therapeutic outcomes [148]. Nanodrug delivery system based nanoparticles have altered the landscape of conventional cancer therapy [145]. TAMs have been targeted using nanodrug delivery systems to enhance the efficacy of breast cancer treatment [43,150]. TAMs can be cured via nucleic acid therapies delivered by nanodrug delivery systems (NDDSs) with a variety of designs [151]. Recent research has revealed that nanodrug delivery methods play a crucial role in TAM-based immunotherapy [152,153]. These systems deliver pharmaceuticals or active chemicals to tumor locations, either directly or indirectly inducing immune responses, as shown in Table 2 [154].

**Table 2:** Drug delivery systems targeting the site of tumor without affecting normal cells.

Nanocarrier	Therapeutic agent	Advantages	Model	Reference
Pegylated polyethylenimine and zymosan complex	DOX and Zymosan	- Reducing toxicity - Promote antiangiogenesis	*In vitro* & *In vivo*	[112]
Hyaluronic acid modified Fe_3_O_4_ NPs	DOX	- Enhance cell phagocytosis -Upregulate M1 macrophage - Downregulate M2 macrophage	*In vitro* & *In vivo*	[155]
LNs	DHA and PTX	- Reprogramming	*In vitro* & *In vivo*	[64]
Cationic polymeric NPs	siCCR2	- Block CCR2 expression in monocytes - Reduce TAMs abundance	*In vitro* & *In vivo*	[156]
Liposome	Zol	- Selective targeting - Repolarization of TAMs	*In vitro* & *In vivo*	[157]
PLGA	Imiquimod and DOX	- Reprogramming	*In vitro*	[158]
PLGA Polymeric NPs	PTX and Trastuzumab	- Improve solubility - Enhance stability - Enhance bioavailability with no issue of hypersensitivity reactions - Blocking the pgp efflux transport protein system	*In vitro*	[159]
Micelles	DOX	- Enhanced cell apoptosis	*In vitro* & *In vivo*	[58]
Chitosan	Oxalipaltin	- Chemically stable - Biodegradable and noncytotoxic	*In vitro*	[160]

PLGA: Poly(lactic-co-glycolic acid); NPs: Nanoparticles; LNs: Lipid nanoemulsions; DOX: Doxorubicin; DHA: Docosahexaenoic acid; PTX: Paclitaxel; siCCR2:Small Interfering and Zol: Zoledronic Acid.

TAMs play critical roles in suppressing the immune system in metastatic breast cancer, and nanodrug delivery devices have been demonstrated to benefit metastatic breast cancer therapy [161]. To improve their selectivity for TAMs, nanoparticles can be functionalized with targeted ligands [105]. Some nanoformulations have been designed to selectively deliver lower dosages of tamoxifen to breast tumors while maintaining high accuracy and reducing off target adverse effects [150]. For the treatment of breast cancer, injectable nanodrug delivery devices have been developed [105,162]. These systems have the potential to increase drug stability, blood circulation time, aqueous solubility, controlled release, and other properties [105,149]. Combination therapies can target various components in TME, including TAMs, the tumor extracellular matrix, the tumor vasculature, and immunosuppressive components [105,149]. Overall, studies on TAM targeting nanodrug delivery systems for breast cancer treatment focus on optimizing drug delivery to TAMs, improving antitumor efficacy, and reducing immunosuppression by modifying the immunosuppressive environment. These approaches offer various advantages and could potentially be used to treat breast cancer.

## 13 Nanocarriers deliver plant-derived therapeutics to modulate TAM

Researchers have explored several plant-derived compounds, such as curcumin, genistein, and resveratrol, for their potential as antineoplastic agents and for loading them into nanocarriers to target TAMs in breast cancer. Curcumin, a polyphenolic component extracted from turmeric, possesses antiinflammatory and anticancer characteristics. Loading it into nanoparticles can improve its bioavailability and targeting capabilities for TAMs [101,150]. Research has shown that curcumin can effectively inhibit the growth of cancer cells at every stage, from initiation to progression, both *in vitro* and *in vivo* [163]. Because it forms bonds with the fatty acyl chains of cell membrane lipids through hydrophobic interactions and hydrogen bonding, it can't get through biological membranes. As a result, curcumin levels in the cytoplasm remain extremely low. A potential solution to these challenges and an increase in bioavailability is curcumin nanosystems, which enhance curcumin's therapeutic properties [163]. Also, genistein is an attractive candidate for anticancer treatment because of its ability to trigger cell cycle arrest, apoptosis, and angiogenesis inhibition. Additionally, it controls epigenetic regulation and modulates multiple signaling pathways, such as the PI3K/AKT and MAPK (ERK1/2) pathways [164,165]. Loading genistein into nanoparticles can improve its bioavailability and targeting capabilities for TAMs. Because of its poor bioavailability and metabolism, genistein may have its limitations circumvented by this approach [166]. Substantial research has also explored the potential anticancer effects of resveratrol, a polyphenolic compound present in many plants [107]. We can load resveratrol into nanoparticles to improve its bioavailability and targeting capabilities for TAMs [9]. Researchers have shown that resveratrol-loaded nanoparticles inhibit the proliferation of breast cancer cells *in vitro*. *In vivo* investigations have demonstrated the ability of these nanoparticles to target TAMs and suppress tumor growth [167]. Combining nanosystems with resveratrol enhances water solubility, stability, and permeability across biological membranes, leading to an enhanced permeation and retention effect (EPR) at tumor sites [167,168]. Overall Different nanocarriers, such as liposomes, polymeric nanoparticles, or metallic nanoparticles, can encapsulate plant-derived compounds to enhance their effectiveness and ability to target TAMs in breast cancer.

## 14 Preclinical studies evaluating the potential of nanomedicines for targeting TAMs

Preclinical studies have revealed remarkable progress in the fabrication of TAM targeted nanomedicines for cancer immunotherapy [3,169]. These nanomedicines propose to overcome challenges associated with TAM induced tumor immunosuppression and increase therapeutic specificity and efficacy [36]. As shown in Table 3, numerous types of nanomaterials have been investigated, including multifunctional nanocarriers with high solubility, cell specific delivery, and controlled release of therapeutic payloads [156,170].

**Table 3:** Recent strategies for TAM targeted nanomedicines in preclinical studies for BC therapy.

Nanocarrier	Target	Therapeutic agent	Effect	Reference
β-Cyclodextrin	TLR7	R848	Re-Education	[171]
Liposome	STING	cGAMP	Re-Education	[104]
PLGA	CD44	Docetaxel	Improved *in vitro* cellular uptake and cytotoxicity	[172]
Liposome	LHRHR	Mitoxantrone	Suppress tumor growth	[173]
PD-L1 Anti-body	PD-L1	TLR agonist	Re-Education	[99]
7C1 nanoparticle	CX3CL1 mRNA	CX3CL1 siRNA	Recruitment Block	[174]
Polymeric micelles	Mannose receptor on TAM	Imiquimod (R837) and DOX	Activation of macrophage Suppress tumor growth	[175]
Liposome	PD-1	DOX	Suppress tumor growth	[176]

PLGA: Poly(lactic-co-glycolic acid); DOTAP: 1,2-dioleoyl-3-trimethylammonium-propane; PC: Phosphatidylcholine; TLR7: Toll-like receptor 7; STING: Stimulator of interferon genes; LHRHR: Luteinizing hormone-releasing hormone; PD-1: Programmed cell death protein 1; cGAMP: and Cyclic guanosine monophosphate-adenosine monophosphate.

Preclinical trials have been carried out to investigate the use of nanotechnology for macrophage targeting in diseases that include various types of cancer and inflammatory disorders [153]. An instance of ongoing clinical research focuses on examining the effectiveness of liposomal nanoparticles containing a chemotherapy drug as a tool for TAM targeting in solid tumors [120,177]. While preclinical models have shown promising results, there is still a requirement for enhancing nanoparticle design and utilizing preclinical models that closely mimic the human environment to validate their effectiveness as precision medicine.

## 15 Conclusion

In conclusion, this review highlights the pivotal role of tumor associated macrophages in breast cancer progression. TAMs are the predominant immune cells in cancerous growths, making up around half of the total immune cell population infiltrating the tumor. These are divided into two separate subgroups, TAM1 and TAM2, which have diverse roles and characteristics. TAM1 displays a proinflammatory phenotype and plays a role in phagocytosis, identification of cancer cells, and initiation of tumor cell killing. The organism generates proinflammatory cytokines and exhibits major histocompatibility complex class II, enhancing antigen display. Conversely, TAM2 plays a role in diminishing inflammation and encouraging fibrosis, which establishes it as the prevailing subtype inside the tumor microenvironment. TAM2 has been linked to the promotion of tumor growth, invasion, and metastasis in breast cancer. An imbalance in the populations of TAM1 and TAM2 might result in the development of pathogenic conditions, such as chronic inflammatory diseases or immune suppression. Hence, approaches aimed at inducing repolarization of TAMs towards the TAM1 phenotype have demonstrated potential in suppressing tumor proliferation. The review emphasizes the promising potential of targeting TAMs for innovative breast cancer therapies, particularly through nanomedicine approaches. Nanotechnology provides potential opportunities for TAMs in the treatment of breast cancer. Nanocarriers, including inorganic, organic, and metals, have been employed to transport chemotherapeutic drugs and gene treatments to breast tumor cells. This approach has resulted in enhanced treatment efficacy and decreased drug resistance. In addition, researchers have created nanocarriers that can react to specific stimuli and modify the tumor microenvironment. These nanocarriers aim to improve the immune system responses to tumors and change the behavior of macrophages. This review underscores the importance of continuing research into TAMs as a means to improve breast cancer treatment outcomes and enhance antitumor immune responses.

## Consent for publication

All the authors read and agreed to publish this article.

## Funding

This work was supported by the 10.13039/501100022230Deanship of Scientific Research, Vice Presidency for Graduate Studies and Scientific Research, 10.13039/501100020912King Faisal University, Saudi Arabia [Grant No. KFU241725 ].

## Data availability

Data included in article/referenced in article.

## CRediT authorship contribution statement

**Ghazala Muteeb:** Writing – review & editing, Writing – original draft, Conceptualization. **Doaa S.R. Khafaga:** Writing – review & editing, Writing – original draft, Conceptualization. **Manar T. El-Morsy:** Writing – review & editing, Writing – original draft. **Mohd Farhan:** Writing – review & editing, Writing – original draft. **Mohammad Aatif:** Writing – review & editing, Supervision, Project administration, Funding acquisition, Conceptualization. **Mohamed Hosney:** Writing – review & editing, Writing – original draft, Supervision, Project administration, Conceptualization.

## Declaration of competing interest

The authors declare that they have no known competing financial interests or personal relationships that could have appeared to influence the work reported in this paper.
